# Supplementary material for: The genome sequence of empress tree, Paulownia tomentosa (Thunb.) Steud. (Lamiales: Paulowniaceae)
Source: Wellcome Open Res. 2026 Sep 21;11:723. [Version 1] doi: 10.12688/wellcomeopenres.27878.1 (PMC13684017; doi:10.12688/wellcomeopenres.27878.1)
Supplement: Peer review history

# Reviewer report: Reviewer response for version 1

**DOI:** 10.21956/wellcomeopenres.30675.r171224

**Published:** 2026-10-20

**Authors:** Oliver Rupp

**Affiliations:** 1 Justus-Liebig-University, Giessengiessen, Germany

This article presents the assembly of Paulownia tomentosa. The data creation and data analyses follow state-of-the-art methods for eukaryotic genome assemblies established at the Darwin Tree of Life Consortium, including PacBio Hifi and Hi-C sequencing. The assembly with hifiasm in Hi-C mode was followed by Hi-C scaffolding and manual curation.

 The quality assessment shows a high quality genome assembly covering all major assembly quality metrics.

 The methods are clearly presented.

Are the datasets clearly presented in a usable and accessible format, and the assembly and annotation available in an appropriate subject-specific repository?

Yes

Are sufficient details of the sequencing and extraction, software used, and materials provided to allow replication by others?

Yes

Are the rationale for sequencing the genome and the species significance clearly described?

Yes

Are the protocols appropriate and is the work technically sound?

Yes

Reviewer Expertise:

genome assembly

I confirm that I have read this submission and believe that I have an appropriate level of expertise to confirm that it is of an acceptable scientific standard.

# Reviewer report: Reviewer response for version 1

**Published:** 2026-09-21

**Authors:** Automated Benchmarking

**Affiliations:** 1 Wellcome Sanger Institute, Hinxton, UK

As this article is a Genome Note, Automated Benchmarking has been used to determine whether the reported assembly passes four defined checks established by the Earth BioGenome Project: the raw and assembled data are publicly available, the Earth BioGenome Project three-part metric is met, the k-mer completeness exceeds 90%, and the BUSCO score passes the stated threshold. For Paulownia tomentosa (daPauTome1), all four checks passed. Data available: pass. ENA study PRJEB87967, raw-read accessions ERR14835597, ERR14835906, ERR14835513, and ERR14835514, and assembly accessions GCA_965263725.1 and GCA_965263755.1 are public. Earth BioGenome Project three-part metric: pass. Haplotype 1 is reported as 7.C.Q64, meeting the 6.C.Q40 reference standard. k-mer completeness: pass. The combined haplotype assemblies have 99.11% k-mer completeness, exceeding the Earth BioGenome Project target of 90%. BUSCO: pass. Haplotype 1, which is proposed as the reference assembly for this species, has 98.9% complete BUSCOs (87.8% single-copy and 11.1% duplicated). BUSCO software version: 5.7.1; lineage dataset: eudicots_odb10. It meets the plant-specific target of greater than 90% complete BUSCOs. This decision uses values reported in the Genome Note and public accession records.

Reviewer Expertise:

NA

I confirm that I have read this submission and believe that I have an appropriate level of expertise to confirm that it is of an acceptable scientific standard.
